# Intersectionality in help-seeking for eating disorders: a systematic scoping review

**DOI:** 10.1186/s40337-025-01202-4

**Published:** 2025-02-13

**Authors:** Jessica Wilkins, Muhammad Ahmed, Karina Allen, Ulrike Schmidt

**Affiliations:** 1 Centre for Research in Eating and Weight Disorders, Institute of Psychiatry, Psychology and Neuroscience, King’s College London, London, UK; 2 South London and Maudsley NHS Foundation Trust, London, UK

**Keywords:** Eating disorders, Help-seeking, Treatment seeking, Intersectionality, Marginalised social characteristic, Stigma

## Abstract

**Background:**

Individuals with marginalised social characteristics (e.g. by race/ethnicity, gender, body weight) report experiencing eating disorder (ED) symptoms but do not proportionally access treatment. There may be unique factors experienced by individuals with multiple marginalised social characteristics which are not included in our current understanding of help-seeking for EDs. The present review sought to examine the extent of evidence exploring help-seeking and service utilisation for (EDs) by people with more than one marginalised social characteristic or identity.

**Main:**

A systematic scoping review was conducted in accordance with the Joanna Briggs Institute guidelines for scoping reviews. Four databases (PsycINFO, PubMED, Embase, Medline) were searched for papers explicitly examining help-seeking or service utilisation for people with more than one marginalised social characteristic or identity (e.g. race/ethnicity, sexual orientation, weight status). Included studies underwent qualitative synthesis employing an existing model of help-seeking adapted for this review. The most common ED investigated was binge eating disorder (BED) and the most frequently reported marginalised characteristics were overweight/obesity and race/ethnicity. Other intersectional characteristics identified included socioeconomic status (SES), gender, and sexual orientation. People with marginalised social identities such as race/ethnicity or gender were more likely to seek help for BED when they also experienced a higher BMI. There was consensus across studies included in this review that help-seeking rates are low for people with an ED.

**Conclusion:**

Mental health literacy and cultural beliefs about help-seeking are important factors affecting the experiences of people with intersectional identities and this may impact their likelihood to seek help. Results suggest that future studies should consider the interaction between social characteristics and identities in their analyses of outcomes in EDs as this is an emerging area of research, extension of our findings is also needed. The protocol for this review is registered via PROSPERO number CRD42024525849.

**Supplementary Information:**

The online version contains supplementary material available at 10.1186/s40337-025-01202-4.

## Abstract (plain language summary)

Eating disorders (EDs) are serious mental health problems but only a few people with EDs access treatment. EDs affect people from all backgrounds, but some groups, like people from ethnic minorities or living with overweight or obesity, have a harder time getting ED treatment. This review examines what existing research tells us about the experiences of people who may face extra disadvantages because they have more than one social characteristic such as their gender or their body size. It includes thirteen studies, all analysed using an established help-seeking model for mental health. The review reports on how often people with EDs seek help, what they think about getting help, the types of help they seek, and how having multiple marginalised social characteristics affects their help-seeking.

**Supplementary Information:**

The online version contains supplementary material available at 10.1186/s40337-025-01202-4.

## Main text

Eating disorders (EDs) are serious mental illnesses affecting at least 7% of U.K. adults [1] and up to 12.5% of adolescents [2]. They significantly impact psychosocial functioning and physical health, and negatively affect the quality of life (QoL) of individuals and their families [3]. Individuals can experience long durations of untreated illness before accessing their first evidence-based treatment; up to 2.5 years for anorexia nervosa (AN), increasing to 4 years for bulimia nervosa (BN) and 6 years for binge eating disorder (BED). This protracted period before accessing treatment negatively impacts outcomes, including the persistence of symptoms [4, 5]. Evidence from the U.K. and Australia demonstrates that early intervention (EI) programs for EDs improve outcomes but only a minority of individuals with EDs access timely treatment [6, 7]. Research exploring delays to accessing treatment has identified both service-related factors and individual factors contributing to delays in help-seeking [8]. Individual characteristics and demographic factors including race/ethnicity, gender, and body mass index (BMI) have all been demonstrated to influence help-seeking [9]. A recent systematic review of the extent of literature on help-seeking for EDs identified that the majority of studies predominantly rely on the experiences of white, cis-gender, heterosexual women. Insufficient attention has been paid to understanding factors which may contribute to help-seeking by individuals from more diverse groups experiencing EDs [8].

Although EDs present across all ethnicities and race groups, disparities exist in diagnosis and access to treatment for some under-served groups [10, 11]. Black and Asian people are less likely than white people to receive a referral for ED treatment although they may be more likely to experience ED symptoms [12, 13]. Other under-served groups who may struggle to access effective treatment include individuals from sexual and gender minority groups [14], individuals of low socioeconomic status (SES) [15], individuals with overweight or obesity [16], and men [17]. Although cis-gender men are not a marginalised social group, they are under-served in ED treatment and experience gender-specific barriers when seeking help for eating problems [18]. Individuals with marginalised social characteristics or identities report experiencing ED symptoms but are not proportionally accessing specialist treatment or represented in research studies. This suggests that there may be unique factors in their experience of help-seeking and accessing treatment which aren’t included in our current understanding of help-seeking for EDs.

Although the term ‘help-seeking’ is frequently used in research, there is no singular, commonly referenced definition. Rickwood and Thomas [19] defined help-seeking for mental health problems as “an active and adaptive process of attempting to cope with problems or symptoms by using external resources for assistance.” Their article reviewing help-seeking research across general mental health highlighted the importance of considering both formal (e.g. speaking to health care providers, specialist treatment) and informal (e.g. friends and family) sources of help-seeking while also distinguishing between processes which facilitate help-seeking (attitudes toward health care) and sources of help by formal, informal and ‘self-help’ descriptions. For the present review, authors adapted Rickwood and Thomas’ [19] model of help-seeking to organise and synthesise data extracted on help-seeking and service utilisation for people with multiple marginalised characteristics (Table 1).

**Table 1:** Model of help-seeking

Category	Features	Example
Influences	Identifying the problem	*Knowledge about ED*
	Mental health literacy	
	Intersectionality	*Factors relating to possessing more than one marginalised characteristic*
Process	Orientation	*Attitude toward help-seeking*
	Intention	*Future planning*
	Behaviour	*Attending treatment appointments*
Time Frame	Ever	
	Specific length	*e.g. last 12 months*
Source	Formal	*Health Care*
	Semi-Professional	*Nutritionist*,* Teacher*,* Clergy*
	Informal	*Friends*,* Family*
	Self-help	*Dieting*,* guided self-help for ED*
Type	Instrumental	*Financial*,* Transportation*
	Information	*Web searches*
	Affiliative	*Peer support*
	Emotional	
	Treatment	*ED Treatment*
Concern	General Distress	*Disordered Eating*
	Specific Symptoms	*Eating Disorder*

Several existing systematic reviews have focused on understanding help-seeking experiences for single groups under-served in ED research. These include reviews of the experiences of men [17], transgender and non-binary individuals [14], racial and ethnic minority groups [20], and members of the LGBTQ + population [21]. These reviews reported that individuals from these groups report elevated levels of ED symptoms compared to white, heterosexual individuals and yet individuals from these groups are disproportionately under-served in existing research. They also suggest unique barriers to help-seeking experienced by individuals in these groups. For example, gendered understandings of EDs can impact men’s self-recognition of the severity of their illness and contribute to delays in help-seeking. Another frequently cited barrier to help-seeking is stigma and shame, but in relation to men and those who live with overweight or obesity, there is evidence that internalised stigma and shame (e.g. being at a higher weight and experiencing an ED) present unique barriers to help-seeking which may not be addressed by traditional interventions designed to reduce stigma [16, 17, 21]. To date, no systematic reviews have focused on understanding the impact of multiple marginalised social characteristics or have been explicitly informed by current conceptualisations of intersectionality on access to treatment and help-seeking behaviour for EDs.

The concept of intersectionality originates from the work of Black feminist and critical race theorists in the U.S [22]. Intersectionality posits that systemic factors influence how individuals with multiple marginalised characteristics may experience disproportionate risk or discrimination due to the intersection of these characteristics. Previously published scoping reviews have explored the concept of intersectionality in general mental health populations. For example, a scoping review exploring prevalence of depression in U.S. adolescents found intersecting characteristics of race/ethnicity and gender compounded risk across multiple studies [23]. Another scoping review, attempting to synthesise the extent of research on intersectionality across mental health, reported studies to be heterogenous and concluded that there is value in assessing intersectional inequalities in mental health research for improving access to care [24]. It is a newly emerging area of study to consider how intersectionality may influence access to care in EDs with studies reporting compounding risk for individuals with multiple characteristics [10].

## Aims of the review

This paper reviewed existing ED research on help-seeking which has considered intersectionality of multiple marginalised social characteristics, with a view to answering the following questions:

1. What is the extent of the research evidence exploring help-seeking or service utilisation by individuals with multiple marginalised social characteristics or identities?
2. What are the characteristics of these studies?
3. What is the existing evidence about help-seeking experiences of individuals with intersectional characteristics?

## Methods

We conducted a systematic scoping review to examine existing research exploring intersectionality of multiple marginalised social characteristics and help-seeking for EDs. This methodology was deemed appropriate because of the emerging and varied nature of research exploring intersectionality in EDs as well as the heterogeneity of methodology in research into help-seeking for EDs. Scoping reviews include a broader range of evidence sources and aim to investigate the extent and nature of existing research in diverse fields. This review was conducted in accordance with the guidelines for scoping reviews developed by the Joanna Briggs Institute [25] and the PRISMA statement guidelines for scoping reviews [26].

### Search strategy

An initial limited search of PsycINFO and MEDLINE was undertaken to identify articles on help-seeking for EDs and intersectionality of marginalised characteristics. The text words contained in the titles and abstracts of relevant articles, and the index terms used to describe the articles were used to develop a full search strategy. The search strategy, including all identified keywords and index terms, was adapted for each included database and information source. On 22 March 2024, a review of PsycINFO, MEDLINE, Embase, and PubMed was run, and the results uploaded to the review software Covidence for selection. For completeness, a web search using Google Scholar and a manual screening of reference lists was also conducted and selected articles added to Covidence for review.

### Eligibility criteria

Articles were included in the review if they met both of the following criteria:

1. Study explored help-seeking or service utilisation for individuals with EDs or disordered eating.
2. Study considered the experiences of individuals with more than one marginalised social characteristic (e.g. race/ethnicity, social class, sexual orientation, gender characteristic, weight status).

Articles examining help-seeking solely in relation to EDs or disordered eating for participants with one marginalised social characteristic (e.g. race/ethnicity) were excluded. Similarly, articles exploring intersectionality of multiple marginalised social characteristics and the experience of EDs without explicitly exploring issues related to help-seeking or service utilisation were also excluded. We included research on clinical eating disorders or disordered eating behaviours, relying on individual research studies to define the latter due to the lack of consistent definition of ‘disordered eating’ across studies. Additionally, studies examining help-seeking by individuals with overweight explicitly for weight loss, without seeking help for an eating disorder, were excluded as they fall outside the scope of this review.

### Study selection

**Fig. 1:**
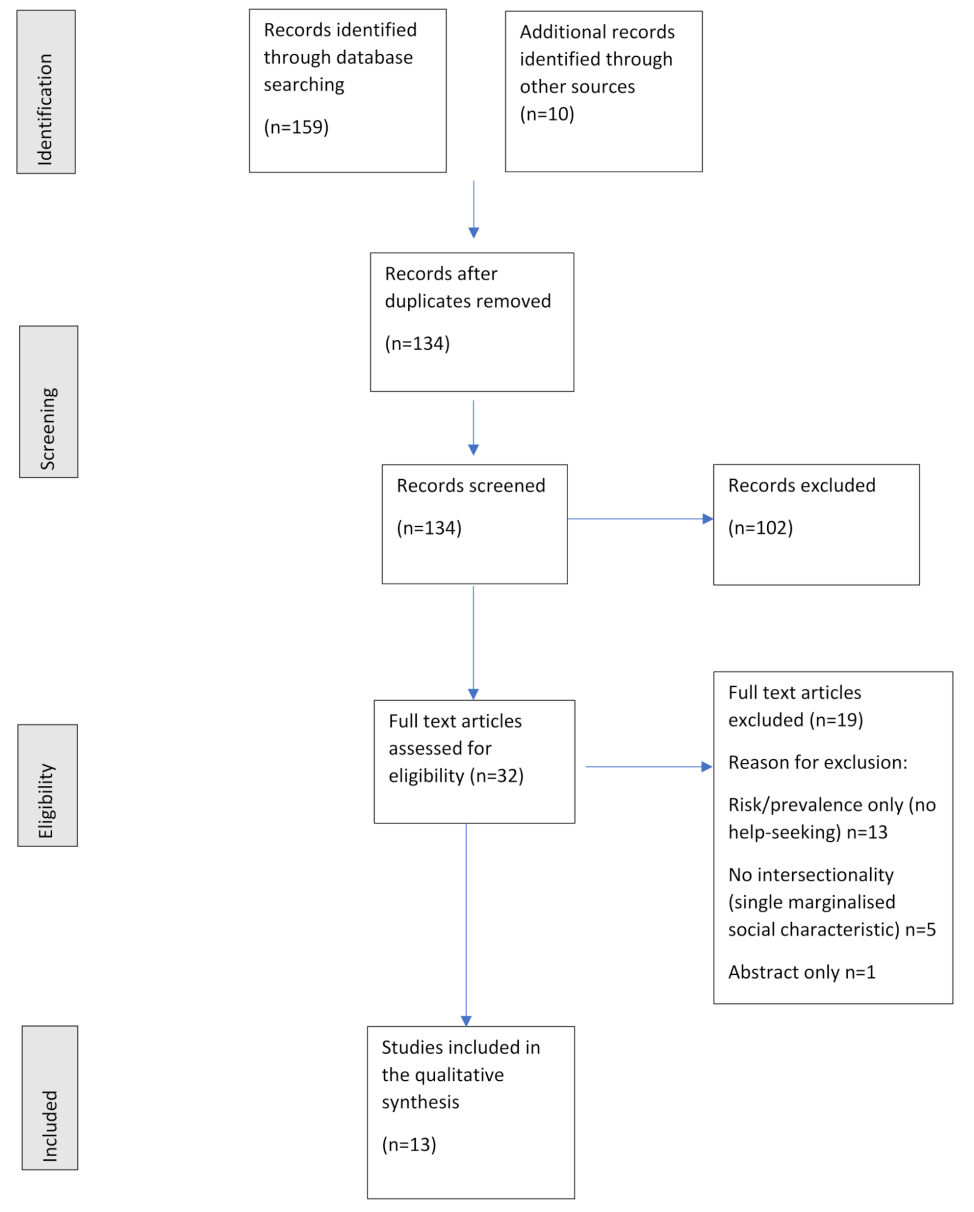
Prisma flow chart

The systematic literature search yielded a total of 134 records following removal of duplicates. Titles and abstracts elicited from the search were screened by two reviewers (JW, MA) independently. JW and MA then randomly selected a sub-selection of 10 articles for comparison to ensure consistency and reduce selection bias. The full texts were then retrieved and assessed against eligibility criteria, with any disagreements resolved through discussion between reviewers until consensus was achieved. Thirty-two full text articles were assessed for eligibility and nineteen of these articles were excluded as not relevant with the reason for their exclusion recorded. A summary of the PRISMA flow chart can be found in Figure 1. Data were extracted into an excel spreadsheet created for the review by JW and checked for accuracy by MA. This data extraction tool, developed for the purpose of this review using an Excel spreadsheet, was used to classify the studies according to themes and organise relevant information about the study (e.g. region of the study, type of experimental design, which marginalised social characteristics examined). All studies selected for inclusion were appraised using the appropriate JBI appraisal checklists by two independent reviewers (JW and MA) who reviewed each checklist together to ensure consensus. No studies were excluded on this basis [27].

## Results

### Study characteristics

This scoping review identified thirteen articles investigating help-seeking or service utilisation for participants with more than one marginalised social characteristic. The majority of studies (*n* = 8, 61.5%) employed a cross-sectional quantitative research design, with a sample of participants accessing clinical services, in the USA (summary in Table 2). The most frequently studied ED in this review was BED (*n* = 10, 76.9%) and of these studies, 60% (*n* = 6) investigated BED only. The most common marginalised social characteristics were weight status (participants with overweight or obesity) and race/ethnicity.

**Table 2:** Summary of Key features of included studies

Characteristic	Number of Studies	
Publication Year		
<2004		1 (7.7%)
2011 − 2006		0 (0.0%)
2017 − 2012		5 (38.4%)
2023 − 2018		7 (53.8%)
Region		
USA		10 (76.9%)
U.K.		1 (7.7%)
Australia		2 (15.4%)
Recruitment Setting		
Clinical		10 (76.9%)
Community		3 (23.1%)
Marginalised Intersecting Social Characteristics Examined		
Weight Status; Race/ethnicity		7 (53.8%)
Gender; Race/Ethnicity		5 (38.5%)
SES; Race/Ethnicity		6 (46.2%)
Weight status; Gender		5 (30.8%)
Type of Study		
Qualitative		3 (23.1%)
Cross-Sectional		8 (61.5%)
Review		1 (7.7%)
Case Series		1 (7.7%)
Measure of Eating Disorder		
EDE-Q		4 (30.8%)
WHO CIDI		2 (15.4%)
SWED		2 (15.4%)
Self-report symptoms		2 (15.4%)
SCID		1 (7.7%)
Type of Eating Disorder		
BED		10 (76.9%)
AN		4 (30.8%)
BN		4 (30.8%)
OSFED		2 (15.4%)
PD		3 (23.1%)
ARFID		2 (15.4%)
OSFED or UFED		2 (15.4%)
“Eating Disorder”		3 (23.1%)

Note. *N* = 13. Not all categories add to 100% as some studies have

more than one characteristic. BED = binge eating disorder, AN = anorexia nervosa, BN = bulimia nervosa, OSFED = other specified feeding or eating disorder, PD = purging disorder, ARFID = avoidant/restrictive food intake disorder, UFED = unspecified feeding or eating disorder

### Narrative synthesis

#### Rates of help-seeking

In keeping with existing research on help-seeking for EDs, measures of help-seeking across studies included in this review were heterogenous and are summarised in Table 3. In addition, definitions of ‘disordered eating’ and measurements of ED also varied. Generally, studies referred to ‘disordered eating’ as a range of behaviours or attitudes towards eating and body image which negatively impacted a participant but may not meet diagnostic criteria for an ED. Similarly, measures of ED diagnosis also varied, but most often included elements of self-report from participants (e.g. by EDE-Q) and interview questions from researchers (e.g. WHO Composite International Diagnostic Interview).This limits ability to make direct comparisons across studies examining rates of help-seeking for people with marginalised social characteristics. Studies predominantly relied on participant self-report of “ever” seeking help/support from a health professional. Usually designed as a single dichotomous question, all studies focused on whether participants had accessed formal help from a health care professional. Examples of informal sources of help-seeking assessed were “social support”, “help from non-mental health professionals” [28] and the use of self-help resources related to dieting [29]. Overall, across studies which reported rates of help-seeking in their samples (Table 4), help-seeking was reported to be low compared to levels of disordered eating or ED diagnosis, which is consistent with established literature on help seeking for people EDs [9]. In one sample, 90.5% of participants reported never accessing mental health support for eating problems [29]. In a longitudinal follow up study of individuals who met criteria for an ED at baseline, only 37.2% of females and 23.6% of males endorsed seeking treatment at 3-month follow-up although authors of this study did not report whether they asked participants about biological sex or gender [30].

Of the studies in this review which reported prevalence of help-seeking, 63% (*n* = 5) found that individuals with multiple marginalised characteristics reported lower rates of help-seeking compared to a control group of participants with one marginalised characteristic or non-marginalised controls [30–34]. Conversely, a minority of studies found that, among participants with one marginalised social characteristic, the addition of a second marginalised characteristic did not significantly impact help-seeking rates. In a sample of adolescents in the community, individuals who had emigrated to Australia were most likely to report seeking help for eating problems but this was not associated with sex, sexual orientation, BMI or SES [35]. Similarly, in a study of gender and help-seeking for an ED, researchers found that men were less likely to seek help-compared to women, but across men there was no difference in likelihood to seek help related to BMI [32].

**Table 3:** Measures of help-seeking

Authors	Help-Seeking Behaviour	Time-Frame	Source	Type
Coffino, et al. (2022)	Weight loss, dieting or “psychologist/psychiatrist/other mental health”	Ever	Formal and Semi-formal	Treatment or weight loss
Lee-Win, Mendelson, Motjabai, (2014)	“Have you ever talked to a doctor or other health professional about eating or weight problems”	Ever	Formal	Not-specified
Tsong et al., (2022)	“Did you seek help for ED-related issues”	Ever	Not-specified	Not-specified
Laboe et al. (2023)	Current or past treatment, “yes/no”	Ever	Formal	Treatment
Grilo et al. (2004)	BED Treatment study attendance	Present	Formal	Treatment
Thapliyal et al. (2020)	“Have you spoken to/sought advice from a professional person specifically in relation to a problem with your eating”	Not specified	Semi-formal	Not specified
Sonneville & Lipson (2017)	Treatment	12 months	Formal	Treatment
Franko et al. (2012)	BED Treatment study participation	Present	Formal	Treatment
Fatt et al. (2020	Sought help from a professional	Ever	Formal or Semi-formal	Treatment
Grammer et al. (2022)	Treatment from a physician, counsellor or other health care provider	Past 2 months	Formal	Treatment

**Table 4:** Help-seeking rates reported

Study	Sample	Measure of Help-Seeking	Rate Reported
Coffino, J.A. et al. (2022)	Clinical, Adults	Weight loss program or “psychologist/psychiatrist/other mental health”	90.5% of participants reported never accessing mental health support for eating problems. “Self-help diets” were most commonly reported “help-seeking”
Lee-Win, A., et al. (2014)	Community, Adults	“Have you ever talked to a doctor or other health professional about eating or weight problems”	Asian Americans less likely to seek help compared to non-Latino white people. Asian American men less likely to report help-seeking compared to white men. Rates not reported.
Tsong, Y. et al., (2022)	Community, Adults	“Did you seek help for ED-related issues?”	14.5% of entire sample reported previous/current treatment
Thapliyal, P. (2020)	Community, Adults	“Have you ever spoken to/sought advice from a professional person specifically in relation to a problem with you eating”	8.47% of participants had ever sought treatment for an ED. Of those who reported seeking treatment, men less likely to seek help compared to women.
Sonneville, R., et al. (2017)	Community, Adults	“Have you ever been diagnosed…” or had treatment in past 12 months for ED.	For entire sample: 13.6% perceived need for treatment 10.5% diagnosed 13.6% received treatment
Fatt, S.J., (2020)	Community, Adolescents	“Have you ever sought help from a professional”	10.1% of those with ED reported ever seeking help. Emigrants more likely to seek help but not significantly associated with sex, sexuality, BMI or SES.
Grammer A.C., et al. (2022)	Community, Adults	“In the past two months have you sought treatment from a physician, counsellor, or other health care provider for an ED or associated weight problem?”	37.2% of females and 23.6% of males endorsed treatment seeking at follow up.
Laboe, A. et al. (2023)	Community, Adults	“Are you currently in treatment for an ED” “Do you intend to seek professional help/and or take any steps to address these concerns”	55.8% reported intention to seek help; 3.09% reported current treatment, 9.46% reported previous treatment

### Help-seeking process

Rickwood and Thomas [19] identified three parts of the help-seeking process in their model of help-seeking for mental health problems. The help-seeking process includes a person’s attitude/orientation towards seeking help, their intention to seek help and finally the help-seeking behaviour itself. Most articles in this review focused on help-seeking behaviour by investigating participant self-report of help-seeking from a health professional, either at some time in the past or currently. Some articles examined participants’ intention for future help seeking, for example, by asking whether participants would use a digital intervention program for BED if it later became available [36]. Two studies asked participants both about their intention to seek help in the future as well as current help-seeking behaviour by engagement in treatment. In these studies, more respondents indicated an intention to seek help in the future than had received help [33, 37]. In survey respondents with probable EDs, 55.8% reported intention to seek treatment but only 12% reported being currently or previously treated for an ED [38]. In a study of Asian American women’s experiences of help-seeking for eating problems and body image disturbance, researchers explored both barriers and facilitators to seeking support. They found that individual attitudes and orientations toward help seeking existed within cultural norms and beliefs. Participants expressed beliefs that “disordered eating was a personal weakness” and described difficulty accepting it as a problem. While these attitudes toward help-seeking have been cited in previous research exploring barriers to help-seeking in ED, authors of this study suggested that these barriers were intensified in the context of cultural norms and beliefs related to their Asian American community [28].

### Source and type of help-seeking

The majority of studies included in this review explored formal and semi-formal help-seeking sources through treatment or speaking to specialist ED or general health professionals. In studies which asked about informal sources of help-seeking, participants with one marginalised social characteristic were more likely to endorse this type of help-seeking compared to formal support [28]. This is in keeping with previous research on help-seeking in individuals from under-served groups in ED [39, 40]. Among participants experiencing BED, overweight, and food insecurity, authors identified the need for instrumental forms of support (e.g. financial, transportation) in making an accessible digital intervention for the treatment of BED, for example, by making the application free or low cost and including a range of budgets in meal planning [36]. Informational forms of help-seeking were referenced in studies with participants with BED and overweight, with frequent reference to self-help dieting educational resources [29, 36]. In keeping with previous research about the utilisation of primary care services, Fatt et al. [35] reported that adolescents in their sample identified as most likely to seek care from a GP rather than a specialist health care professional. No studies reported whether there was a difference in type or source of help-seeking for individuals with marginalised social characteristics.

### Intersectionality

All studies included in this review, in line with the eligibility criteria, examined the influence of multiple marginalised social characteristics on participant help-seeking as part of their study. A number of studies found no significant differences when comparing the experiences of participants with multiple marginalised characteristics to white participants or those with only one marginalised social characteristic [35]. Other studies included data on participants with intersectional identities; however, they did not perform statistical analyses to determine whether participants with intersectional characteristics had significantly different help-seeking compared to others in their sample [33]. Across all studies there was a theme of mental health literacy, and cultural beliefs about EDs, as a barrier to help-seeking [28, 41]. Beliefs about EDs and mental health literacy has been identified as a barrier to help-seeking for EDs across all populations, but these studies evidence a unique experience for people from minoritised ethnicities [9].

### Weight status

Across all studies examining help-seeking for BED, investigators consistently reported that help-seeking was associated with higher BMI. Two studies in this review examined intersectional identities of weight status and ethnicity on help-seeking for BED. Both studies reported an association between higher BMI and help-seeking in their samples. They also noted that Black women in their treatment-seeking sample possessed a higher level of education, and thus perhaps also higher SES, than those in their communities [34, 42]. Another study, accounting for both ethnicity and SES/BMI, did not explain differences in reported levels of ED symptoms (EDE-Q) but reported participants with higher BMI were more likely to seek treatment. The role of higher BMI and its impact on help-seeking was replicated in a study using a non-treatment seeking community sample, where men were less likely to seek help for an ED. Among those who reported symptoms which met threshold for an ED diagnosis, there was no significant difference in gender and help-seeking. The sample in this study had a higher proportion of participants meeting threshold for diagnosis of BED (compared to other EDs such as AN or BN) and higher BMI was the only significant predictor of help-seeking in this sample [32].

### Ethnicity

In a community sample of adults, Asian Americans were less likely compared to non-Latino white participants to seek help. Asian men were more likely to report history of binge eating that did not meet criteria for BED diagnosis compared to non-Latino white men in the sample, but less likely to seek help. Asian American are less likely to be diagnosed with BED and authors in this study suggested this is due to lower endorsement by Asian American participants of “loss of control” features of BED. This may be an example of how ethnicity-specific factors play a role in symptom endorsement contributing to less frequent diagnosis of ED for people in these groups [31]. Similar results were presented in a qualitative study examining barriers to help-seeking among individuals in the South Asian community, where participants identified that South Asian men may experience significantly more shame and stigma related to ED symptoms and suggested that this may further delay treatment seeking among people in this group [41].

### Socioeconomic status

Two studies in this review examined the impact of food insecurity on help-seeking for an ED. Food insecurity is defined as inadequate or inconsistent access to food and can be operationalised as a proxy for SES, although it is important to note that food insecurity impacts people across the spectrum of SES. In one study, food insecurity was not correlated with treatment status or treatment seeking, however, there was a significant positive correlation between food insecurity and ED symptoms [38]. Among participants who experienced both food insecurity and overweight or obesity, lack of access or affordability of treatment were identified as barriers to accessing treatment. This was conceptualised as compounding shame and stigma from experiencing low SES and concerns regarding weight/shape [36]. Other studies looked more broadly at SES variables including level of education and household income. In a community sample of university students, researchers found that SES did not predict ED diagnosis but was correlated with lower perceived need for treatment and lower treatment engagement. Similar findings were reported for men in the study, who were less likely to report a perceived need for treatment or prior ED treatment. However, the authors did not investigate whether SES could explain any differences observed among men in their sample [33]. And in a study with a community sample of Asian American women, 20% of respondents identified financial resources as significant barriers to seeking treatment [28]. There is evidence from this review of multiple impacts of SES, race/ethnicity and food insecurity on both eating disorder symptoms and barriers to help-seeking.

## Discussion

Intersectionality and EDs is a newly emerging area of research. The results of this review demonstrate that help-seeking for ED is low, and there remains a lack of consensus on how multiple marginalised social characteristics impacts help-seeking and service utilisation. Overall, the majority of studies included in this review examined help-seeking experiences for participants with overweight from minoritised ethnicities with BED. This is important given that individuals with overweight or obesity are often reported to be the least likely to access treatment for ED, alongside evidence that people from minoritised ethnic groups are also among those least likely to seek help or access treatment [16, 43]. It offers evidence that research exploring the unique experiences of people with intersectional characteristics is beginning with those who are most vulnerable or experiencing the greatest barriers to seeking help. In all the studies included in this review that focused on individuals with BED, it was consistently reported that participants seeking treatment had a higher BMI compared to those who did not seek treatment. Participants who reported help-seeking for their ED had a higher BMI compared to those who did not and reported greater eating and shape concerns compared to community samples or participants who had not sought help. Importantly, these correlations were not always associated with higher levels of ED symptoms (for example, number or frequency of binge eating episodes). In a study examining the characteristics of participants in treatment-seeking for BED, Grilo et al. [42] found that Black women seeking treatment reported less frequent binge episodes and lower scores on EDE-Q, but had higher BMI and concerns regarding their weight and shape. This suggests that for Black women with overweight or obesity, their motivation to seek treatment is not always related to the severity of their ED symptoms. Instead, as suggested by another study, distress related to body image or stigma related to weight status may motivate people to seek treatment for their ED [44].

Even when researchers identified participants with more than one marginalised social characteristic, they did not always examine possible compounding or interactional effects of multiple marginalised characteristics on help-seeking for ED. Research beginning to utilise the concept of intersectionality and examining its impact on individual experiences of ED has found that individuals with intersectional identities are more likely to experience ED symptoms and less likely to access services [43, 45]. This suggests it is an important area of enquiry if seeking to understand the experiences of people who are traditionally under-served in ED research. A large number of studies (*n* = 13, 68% of all studies excluded) which examined the impact of intersectional identities on individuals with ED only reported on risk or prevalence of ED among people in these groups. This means that early emerging research on intersectionality and ED currently focuses on how it may impact a person’s risk of developing an ED but not yet on their experience seeking help or service utilisation. Furthermore, it also does not yet examine treatment engagement or outcomes for ED. This would be an important direction for future research to better engage people who may be more likely to experience ED but who are less likely to access specialist treatment in a timely fashion.

Weight, shape, and appearance are part of our social identity and important considerations in help-seeking for ED. Weight stigma exists among both primary care practitioners and ED specialist health care professionals [46, 47]. Combined with differences in aetiology and clinical presentation by individuals with multiple marginalised characteristics there is a risk that these individuals may be misunderstood or under-diagnosed by clinicians. Individual factors unique to members of some marginalised social groups, such as stigma/shame related to mental health and being less likely to recognise their ED behaviours as problematic, may contribute to delayed help-seeking and difficulty in accessing specialised services for ED.

### Study strengths and limitations

*One strength of the current review is that it is the first to explore how intersectionality of multiple marginalised social characteristics influences help-seeking for ED. A second strength is that it links emerging literature in this area to a well-established theoretical model of help-seeking for mental health problems.* Existing studies examining intersectionality and ED have predominantly focused on reporting risk and prevalence for individuals with multiple marginalised social characteristics, with a lack of focus on how these identities may impact help-seeking, service utilisation and treatment efficacy. Furthermore, in studies which collected data on more than one marginalised characteristic for participants, many did not perform statistical analyses to ascertain interactions between multiple marginalised social characteristics and research outcomes including service utilisation or help-seeking. It would be useful for researchers, especially those interested in the experiences of traditionally under-served groups in ED, to consider the interaction between social characteristics and identities in their analysis of outcomes as this area of research emerges.

Additionally, there are several methodological concerns regarding existing studies. Help-seeking is not consistently operationally defined across studies. Most studies rely on self-report from participants and focus on formal help-seeking from health professionals. This may be problematic when we are considering the experiences of people less likely to seek help from specialised ED services and those more likely to utilise informal support such as self-help, social support and the internet. Multiple studies in this review focused on the experiences of people accessing treatment for BED and used seeking weight loss treatment to operationalise help-seeking for ED. While it is important to understand the type of help that people with BED engage with, behavioural weight loss is not synonymous with BED treatment, and is less effective in reducing ED symptoms compared to evidence-based psychological treatments such as CBT [48]. Many studies focused on the experiences of individuals with ED who had accessed treatment, either by participation in clinical trials or specialised ED treatment. If we accept that people from ethnic/racial, gender and sexual minority groups are less likely to seek treatment for their ED we need to consider whether existing research evidence relying on the experiences of treatment-seeking individuals is representative of under-served groups as whole. While participants who do seek treatment may be able to contribute useful understanding to the facilitators of seeking help-seeking for ED, we also need to include the experiences of those who do not find their way into specialised treatment.

Quantitative methodologies were predominantly used in the studies included in this review, with a lack of qualitative methodologies. While quantitative methodologies are useful when research variables are clearly defined, qualitative methodologies offer unique insight when studying complex or nuanced phenomena. Given that intersectionality in ED is one such complex phenomenon, it is likely that qualitative methodologies could make a significant contribution to our understanding of the experiences of people with ED possessing multiple marginalised social characteristics.

Finally, given the newly emerging and heterogenous nature of research on intersectionality of identities and help-seeking for ED, eligibility for the current study was broad to include a number of marginalised social characteristics including, race/ethnicity, gender, sexual orientation, social class, and weight status. Given the unique experiences of individuals from these groups, it would be inappropriate to infer that the experiences of individuals from one group (e.g. individuals from minoritised ethnicities with overweight/obesity) are similar to that of a separate minoritised group by virtue of them both being minoritised. Indeed, the results of this study demonstrate that the experiences of people from these groups vary and it is not possible to draw conclusions about the experiences of all people who may have intersectional identities and live with an ED. Despite this limitation, we believe that this exploration of existing research and subsequent synthesis has enriched understanding of how individuals from under-served groups in ED research approach help-seeking for EDs with the view to direct future investigations in this domain.

## Conclusion

Determining the factors that impede or facilitate help-seeking for EDs is critical in addressing this serious mental illness. Clearly more research is required to better understand these factors, particularly among people with marginalised social characteristics, including those from ethnic/racial minority groups, elevated BMI, low SES status, and from sexual/gender minority groups. The results of this review demonstrate there is value in understanding intersectional inequalities in mental health to improve access to care for under-served groups. Meanwhile, awareness and prevention programs should focus on including the experiences of individuals from these groups with the view to reduce stigma and shame around EDs, and improve education on these illnesses, their impact, and available resources for help.

## Electronic supplementary material

Below is the link to the electronic supplementary material.

Supplementary Material 1: Search strategy

Supplementary Material 2: Data extraction instrument

## Data Availability

No datasets were generated or analysed during the current study.

## Abbreviations

ED: Eating disorder
AN: Anorexia nervosa
BN: Bulimia nervosa
BED: Binge Eating Disorder
ARFID: Avoidant Restrictive Intake Disorder
PD: Purging Disorder
OSFED: Otherwise specified feeding and eating disorder
UFED: Unspecified feeding and eating disorder
BMI: Body Mass Index
