# Supplementary material for: Intersectionality in help-seeking for eating disorders: a systematic scoping review
Source: J Eat Disord. 2025 Feb 13;13:26. doi: 10.1186/s40337-025-01202-4 (PMC11827232; doi:10.1186/s40337-025-01202-4)
Supplement: Supplementary file 1 — Supplementary Material 1: Search strategy [file 40337_2025_1202_MOESM1_ESM.docx]

Intersectionality Scoping Review

Search Strategy

PubMed

(Help seek* OR Seek* help OR Seek* treatment OR health services accessibility [MeSH]) AND

(anorex* OR bulimi* OR “eating disorder*” OR “disordered eating” OR binge* OR eating disorders [MeSH])

AND

(men [MeSH] OR transgender OR bisexual* OR gay OR lesbian* or "sexual minorit*")OR (ethnic minorities [MeSH] OR “multi-ethnic*” OR Black people [MeSH] OR Asian [MeSH] OR Middle Eastern people [MeSH] OR Indigenous peoples [MeSH] OR Hispanic [MeSH] OR “mixed ethnic*”) OR (“low socioeconomic” OR “social class” OR “food insecurity*” OR “intersectional*”)

OVID

(eating disorder* or eating distress* or eating difficult* or disordered eat* or feeding disorder or feeding difficulty or rumination disorder or rumination syndrome or night eating syndrome or purg* or anorexi* or bulimi* or bing* or BED or Pica or allotriophagy or geophag* or Avoidant restrictive food intake disorder or ARFID or UFED or EDNOS)

AND

(help-seek* or seek help or treatment seek* or care seek* or service utili?ation or treatment access*)

AND

(men or male or boy or gender* or transgender* or gender min* or non-binary) OR ethnic minorit* or racial or race or multi-ethnic or black or Middle Eastern or Asian or BIPOC or BAME or Aboriginal* or First Nation or Latin* or marginali?ed or CALD or mixed or immigra* or migrant* or refugee* or gypsy^ or traveller) OR (LGBT* or gay or lesbian* or queer or bisexual) OR (socioeconomic status* or SES or low income or food insecure or food insecurity or homeless*)
