# Supplementary material for: Intersectionality in help-seeking for eating disorders: a systematic scoping review
Source: J Eat Disord. 2025 Feb 13;13:26. doi: 10.1186/s40337-025-01202-4 (PMC11827232; doi:10.1186/s40337-025-01202-4)
Supplement: Supplementary file 2 — Supplementary Material 2: Data extraction instrument [file 40337_2025_1202_MOESM2_ESM.docx]

Adapted from Rickwood & Thomas (2012)

HELP-SEEKING

“help-seeking is an active and adaptive process of attempting to cope with problems or symptoms by using external resources for assistance.”

Source

Formal (health care)

Semi-formal (professional relationship but not health)

Informal (friends/family)

Self-help

Process

Orientation -attitude toward seeking help

Intention – future intention

Behaviour -observable behaviour

Concern

*General distress*

*Specific symptoms*

Influences

identifying the problem or MH literacy

Inter-

sectionality

Type

*Instrumental* (e.g. financial, transportation

*Information* (e.g. web searches)

*Affiliative* (e.g. peer support)

*Emotional*

*Treatment*

Time Frame

Ever?

Specific length (e.g. 12 months)
